# Metal Nanoparticles for Photodynamic Therapy: A Potential Treatment for Breast Cancer

**DOI:** 10.3390/molecules26216532

**Published:** 2021-10-29

**Authors:** Liang Shang, Xinglu Zhou, Jiarui Zhang, Yujie Shi, Lei Zhong

**Affiliations:** 1 Department of Breast Surgery, Second Affiliated Hospital of Harbin Medical University, Harbin 150086, China; shangliang0825@163.com (L.S.); zhangjiarui10200@126.com (J.Z.); or drlanyan@126.com (Y.S.); 2 Department of PET/CT Center, Harbin Medical University Cancer Hospital, Harbin 150081, China; zhouxinglu@hrbmu.edu.cn; 3 Department of Breast Surgery, Sixth Affiliated Hospital of Harbin Medical University, Harbin 150086, China

**Keywords:** breast cancer, photodynamic therapy, metal nanocarriers, synergistic therapies

## Abstract

Breast cancer (BC) is the most common malignant tumor in women worldwide, which seriously threatens women’s physical and mental health. In recent years, photodynamic therapy (PDT) has shown significant advantages in cancer treatment. PDT involves activating photosensitizers with appropriate wavelengths of light, producing transient levels of reactive oxygen species (ROS). Compared with free photosensitizers, the use of nanoparticles in PDT shows great advantages in terms of solubility, early degradation, and biodistribution, as well as more effective intercellular penetration and targeted cancer cell uptake. Under the current circumstances, researchers have made promising efforts to develop nanocarrier photosensitizers. Reasonably designed photosensitizer (PS) nanoparticles can be achieved through non-covalent (self-aggregation, interfacial deposition, interfacial polymerization or core-shell embedding and physical adsorption) or covalent (chemical immobilization or coupling) processes and accumulate in certain tumors through passive and/or active targeting. These PS loading methods provide chemical and physical stability to the PS payload. Among nanoparticles, metal nanoparticles have the advantages of high stability, adjustable size, optical properties, and easy surface functionalization, making them more biocompatible in biological applications. In this review, we summarize the current development and application status of photodynamic therapy for breast cancer, especially the latest developments in the application of metal nanocarriers in breast cancer PDT, and highlight some of the recent synergistic therapies, hopefully providing an accessible overview of the current knowledge that may act as a basis for new ideas or systematic evaluations of already promising results.

## 1. Introduction

Breast cancer (BC) is the most commonly diagnosed cancer that affects women around the world, with more than 1.3 million individuals diagnosed each year [1]. The incidence of breast cancer ranks first in female malignant tumors, which seriously threatens the physical and mental health of women [2]. Management of breast cancer is multimodal, including surgery, chemotherapy, radiotherapy, endocrine, and targeted treatment [3,4,5,6,7]. Although the traditional treatment of BC has been developing, severe side effects or accelerated aggravation always decrease the quality of life of patients [8]. Besides, poor diagnosis of dense breast tissue can lead to tumor recurrence or metastasis, which delays timely treatment and cause higher mortality. Therefore, it is urgent to identify a new adjuvant therapy that can not only lower toxic effects but obtain targeted therapeutic outcomes.

Currently, photodynamic therapy (PDT) is becoming a mainstream cancer treatment, received tremendous attention in the past decades [9]. PDT is a technique for the treatment of malignant tumors, which is a modern and non-invasive form of therapy by utilizing harmless light to activate photosensitive chemicals to generate cytotoxic species for malignant cell eradication [10,11]. First, a drug that absorbs light in the treatment window (650–850 nm), where the tissue is more transparent, is given to the organism. After some time, the target tissue is irradiated. The drug is inactive in the dark; however, when it is excited by electrons, it generates reactive oxygen species (ROS) locally. ROS can trigger three primary biological mechanisms that make PDT an effective anticancer method: vascular closure and massive ischemic death of tumor tissue, a direct killing of tumor cells induced by intracellular oxidative stress, and PDT induces acute local and systemic inflammation, eventually stimulating T cell activation, producing antitumor immune memory and systemic responses [12,13]. Compared with conventional chemotherapy and radiotherapy, PDT is appealing for small trauma, low toxicity, strong selectivity, and broad applicability [14].

The effectiveness and limited side effects of PDT in ablating localized BC tumors is a breakthrough in unconventional treatment [9]. As mentioned above, in the process of PDT, photosensitizers (PSs) will be activated under appropriate light, thereby generating reactive oxygen species, and then destroying cancer cells [15]. However, due to the hydrophobicity of most PSs, they are easily aggregated in aqueous solutions, thereby reducing the effectiveness of PDT treatment [16]. In addition, PSs do not tend to selectively bind to tumor cells, resulting in poor specific uptake of tumor cells, so local normal tissues may be affected during treatment [17]. In this case, the combination of nanotechnology and PDT in the form of a nanoplatform is very important.

Under the current circumstances, nanotechnology has opened up a new era and is now widely used worldwide [18,19]. With the development of nanotechnology, a large number of materials have been researched and developed in the size range of 1 to 100 nm. These materials have considerable potential in biomedical applications [20]. Most nanoparticles (NPs) can be divided into organic (nanoparticles composed of organic materials) and inorganic (physical and chemical properties are attributed to their inorganic components, such as metal or semiconductor materials). Historically, organic nanoparticles, such as dendrimers, micelles, and liposomes, have attracted much attention due to their potential medical applications [21]. Nano-objects synthesized from organic ingredients have been used in many oncology applications with varying degrees of success. Compared with organic nanoparticles, inorganic nanoparticles were developed at the end of the last century, and their biomedical applications are relatively new. All inorganic nanoparticles have a typical core/shell structure. The core can contain metals (iron oxide, gold, and quantum dots) or organic fluorescent dyes encapsulated in silica [21]. The outer shell is usually made of metal or organic polymers, which can protect the core from chemical interactions with the external environment and/or serve as a substrate for binding to biomolecules such as antibodies, proteins, and oligonucleotides. Among inorganic NPs, metal nanoparticles have the advantages of high stability, adjustable size, optical properties, and easy surface functionalization, making them more biocompatible in biological applications [22,23]. Due to these characteristics of metal NPs, its combination with photosensitizers can improve the effect of photodynamic therapy. In addition, the covalent or non-covalent binding of PSs and nanoparticles can improve the active targeting of drugs, so that PSs can be specifically delivered to targeted tumor cells [24].

This review aims to summarize the current knowledge about metal nanomaterials in the photodynamic therapy of breast cancer. First, we briefly discuss the basic principles of PDT in cancer, as they provide a basis for understanding phototoxicity. Secondly, we comprehensively introduce the metal nanomaterials suitable for breast cancer PDT. Finally, we focus on the current preclinical and, in a few cases, clinically, the evidence of the effectiveness of metal nanoparticle PDT as a part of combined therapy for breast cancer.

## 2. Conventional Treatments of Breast Cancer

Currently, chemotherapy, surgery, radiotherapy, endocrine therapy, and targeted therapy are the main treatment methods for breast cancer [25]. Other less invasive treatments, such as cryotherapy, laser ablation, and radiofrequency ablation, have also been developed to treat early breast cancer [26,27]. In recent years, with the continuous improvement of breast cancer treatment methods, the cure rate has been greatly improved. However, it is the biggest obstacle to the successful treatment of breast cancer due to the heterogeneity and high complexity of the disease, as well as insufficient drug concentration to reach the tumor. Under such circumstances, there is an urgent need to seek new and more effective treatment methods to reduce the side effects of treatment and improve the treatment effect of breast cancer.

## 3. Photodynamic Therapy

Photodynamic therapy is a type of light therapy that uses visible light, a photosensitizer, and molecular oxygen to destroy cancer cells [28]. Unlike traditional therapies, PDT has non-invasive and selective cytotoxicity to malignant cells. PDT directly causes tumor cell death through apoptosis, necrosis, and autophagy, which can significantly improve the quality of life and prolong the survival rate of cancer [29,30]. This makes PDT a more attractive form of treatment.

### 3.1. Mechanism of Action

**Figure 1:**
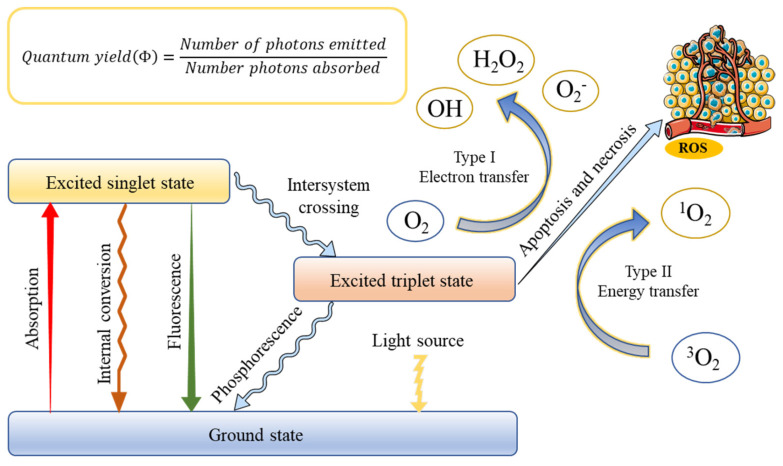
Schematic illustration of a typical photodynamic reaction with the generation of excited states and reactive oxygen species (ROS).

The successful application of PDT requires an optimal combination of a specific wavelength of light, a photoactivated chemical substance called a photosensitizer, and molecular oxygen present in tissues to induce cell death through oxidative damage [31]. The basis of treatment depends on the photosensitizer, which can be absorbed and localized in target cells or tissues, entering the malignant tissue after systemic or local administration [32]. Once the optimal tissue concentration is reached, the tumor tissue can be exposed to low-power visible light for a predetermined time. After the exposure of PS to light containing its action spectrum, it is transformed from the ground state (singlet state) into an electronically excited state (triplet state) [33]. In this triplet state, PS reacts with molecular oxygen to produce reactive oxygen species, which is produced through two pathways [34]. In type I, the PS reacts with biomolecules, through a hydrogen atom transfer, to form radicals that react with molecular oxygen to generate ROS. On the other hand, ions and energy are directly transferred to oxygen to generate ROS, such as singlet oxygen (^1^O_2_), which is called a type II reaction. This production is always regarded as the most crucial process in generating damage during PDT [35]. However, the contribution of the two mechanisms depends on several factors, including oxygen concentration, pH value, and the structure of the photosensitizer. When oxygen is depleted, the first mechanism begins to prevail [36]. The rationale of PDT is shown in Figure 1.

The antitumor effects of PDT mainly involve three mechanisms, including tumor cell death by ROS, the initiation of tumor cell immune response, and tumor-related vascular system damage [37]. PDT causes cell death, usually via apoptosis, necrosis, or autophagy. The interaction of different types of cell death depends on the location of the photosensitizer within the cell. The damage of mitochondria can cause apoptosis, cell membrane damage, and loss of integrity provoke necrosis, and lysosomal or endoplasmic reticulum damage can lead to autophagy [38,39]. Furthermore, the effectiveness of the PDT method is related to the systemic anticancer immune response. PDT destroys the structure of the tumor, thereby stimulating the direct interaction between cancer cells and immune cells. The direct destruction of tumor tissue leads to a robust inflammatory response, and leukocytes infiltrate the tumor, causing a massive release of inflammatory mediators [40]. Cancer cells that did not necrosis in the above process may still be destroyed by the indirect effect of PDT on tumor blood vessels. Activated oxygen damages vascular endothelial cells to activate the coagulation process, aggregates platelets, and blocks blood vessels by forming thrombi, and tumor tissue continues to be hypoxic, resulting in cell death [41]. The effectiveness of PDT largely depends on the choice of PS, which is mainly chosen based on its solubility and selectivity.

### 3.2. Photosensitizers

PDT involves the systemic administration of non-toxic PS, which can accumulate in cells and cause cytotoxicity. Many studies have demonstrated that a wide variety of compounds have been used as photosensitizers in PDT [42,43]. The ideal characteristics of PS include low toxicity, high targeting, and minimal side effects [44].

The usage of PS for therapeutic purposes has started long in the countries like China and Egypt, and eosin is reported to be the first PS for cancer [45]. In the early 1950s, the observation of hematoporphyrin in tumor tissue accelerated the research of new photosensitizers based on porphyrin and its application in cancer treatment. Photofrin and hematoporphyrin are considered to be the first generation of PS [46]. Currently, photofrin is still the most commonly used PS, which has been approved for the treatment of lung cancer, bladder cancer, esophageal cancer, and early cervical cancer [28]. Although it is approved for a wide variety of cancer treatments, it has many drawbacks like low chemical purity, cutaneous phototoxicity, hydrophobicity, and weak absorption in the therapeutic window, which limits its use in clinical treatment [47,48,49].

As early as the 1980s, people began to study the next generation of photosensitizers. The second generation of PSs, which are designed to overcome the undesirable of their predecessors, have promoted PDT in clinical cancer treatment. Hundreds of substances with photosensitive properties have been proposed, of which only a few are used in clinical trials [50]. The second-generation photosensitizers have higher chemical purity, higher singlet oxygen generation rate, and better penetration of deep tissues. Furthermore, they show fewer side effects due to higher selectivity for cancer tissues and faster removal of photosensitizers from the body [51]. The phthalocyanines, which act as a family of compounds, have been investigated for potential use in cancer therapy [52]. Since most of the effective PSs are lipophilic, it is crucial to investigate delivery systems to overcome poor water solubility [53,54]. An example of this is zinc phthalocyanine, which has excellent lipophilic, with good efficacy and no adverse effects in a murine model [31]. Moreover, the cells treated with zinc phthalocyanine decreased viability and proliferation [55]. Zinc phthalocyanine, which can induce programmed cell death in MCF-7 breast cancer, also shows potential as a therapeutic agent when delivered via albumin nanospheres [56].

Currently, PDT is a highly specific therapeutic strategy for killing tumor cells; however, there are still several key issues that need to be resolved. First, due to insufficient oxygen supply in many types of solid tumors, the therapeutic effect of oxygen-dependent PDT will be significantly limited [57,58]. In addition, traditional photosensitizers have poor selectivity and lack of targeting [59]. In order to overcome these limitations of PDT, new photosensitizers are currently being developed, such as nanoenzymes that can catalyze H_2_O_2_ to produce O_2_ to improve hypoxia in tumor tissues, and nano-drug delivery systems that can achieve targeted tumor drug delivery. With the continuous emergence of these new photosensitizers, it can effectively improve the bioavailability of photodynamic methods while maintaining satisfactory therapeutic effects [60].

## 4. Metal Nanoparticles

As we all know, nanomedicine is the medical application of nanotechnology. At present, the use of nanotechnology is becoming more and more popular, and it affects more and more scientific fields [61,62,63,64,65,66]. Richard Feynman introduced the concept of nanoscale particles to the world in 1959 [67,68]. Since then, a lot of research has been conducted in various disciplines to promote the development of this field [69,70,71]. Cancer nanotherapy is an emerging method used to overcome the limitations associated with traditional anti-cancer treatments. The National Cancer Institute (NCI) of the United States has regarded nanotechnology as an emerging field with the potential to create a new paradigm for cancer treatment, and it has already had an impact on prevention and control, early detection, imaging diagnosis, and multifunctional therapy [72]. Current nanomedicine uses fine-structured to target specific tissues and organs. These NPs can be used as antiviral, antitumor, and cancer treatments [73]. These nanomaterials are usually easy to form complex compounds with other substances (including organic materials). At the same time, the developed surface of nanoparticles leads to an increase in their chemical activity, which makes it possible to use oxide nanoparticles to inhibit the growth of pathogenic bacteria, including bacteria resistant to antibiotics [74,75]. Irradiating nanoparticles with relatively low-power radiation can cause light stimulation on the surface of the nanoparticles. This response—especially the production of singlet oxygen—is used in photodynamic therapy. The resulting complex has new characteristics. The development of knowledge of nanoparticle applications has led to the possibility of using this technique in the PDT method [76]. These properties help increase the intracellular concentration of drugs in cancer cells, thereby reducing the toxicity of normal cells. These advantages lead to enhanced anti-cancer activity and reduced systemic toxicity [77].

**Figure 2:**
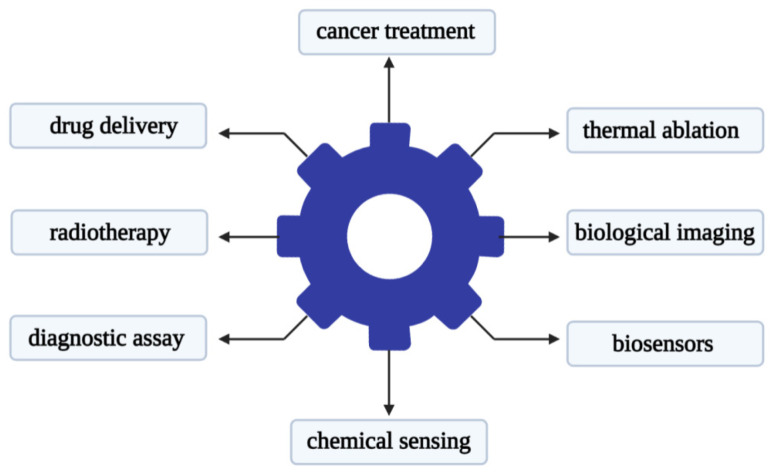
Main applications of gold nanoparticles in the biomedical field.

Recent studies have shown that metal-based nanoparticles can be used as photo-sensitizers, delivery vehicles, and up-conversion tools [78]. Metal nanoparticles have attracted attention in the use of various forms of nano-scale materials. Its advantage lies in its relatively narrow size and shape distribution, and a long activity period [79]. There is reason to believe that metal nanoparticles that are stable in aqueous dispersions will find useful applications in biology and medicine. The use of metal nanoparticles for drug delivery is a promising strategy that can be used to improve the delivery of chemotherapy, radionuclides, and antibody drugs to tumor cells [80]. The research literature on cancer nanotechnology has exploded over the past decade. Gold nanoparticles are one of the most popular materials in metal nanoparticles used in medicine. Gold nanoparticles can be different in size, shape, and structure. Researchers have developed diverse gold nanoparticle formulations for different therapeutic purposes. The main uses of gold nanoparticles are shown in Figure 2.

Gold nanospheres (AuNPs) are formed by the reduction of chloroauric acid. They are solid gold spheres with diameters ranging from a few to more than 100 nm, which can be used for imaging and radiation dose enhancement [81]. One of the most popular synthetic methods to produce gold nanospheres is to reduce tetrachloroauric acid (HAuCl_4_) with sodium citrate water, which was pioneered by Turkevich and further refined by Frens [82,83]. The gold nanospheres produced by this method rely on a multifunctional citrate layer formed on the surface of the nanoparticles, which allows almost any type of molecule to functionalize the resulting particles [84,85]. In addition, there are many methods for preparing citric acid-stabilized gold nanoparticles. Only by changing the reaction parameters (such as the pH of the solution, the order of reagent addition, etc.), monodisperse quasi-spherical particles with a diameter of about 10 to 300 nm can be prepared [86,87]. However, this strategy has not been adapted to produce Au nanoparticles below 10 nm. In recent years, researchers have proposed to mix tannic acid and sodium citrate, two competitive reducing agents, to prepare gold nanoparticles with controllable morphology and a size resolution of 3.5–10 nm, which improves the size of the gold nanoparticles availability of high-quality samples [88].

Gold nanorods (AuNRs) are gold nanorods synthesized from chloroauric acid-containing gold seeds and stabilizers, usually cetyltrimethylammonium bromide [89]. The absorption wavelength of AuNRs has two peaks, which depend on the orientation of the particle to the incident beam. The maximum size of AuNRs is usually 45 nm, and the peak absorption wavelength of these plasma particles is changed by controlling the aspect ratio of these plasma particles [90]. It has been reported that adding benzyldimethylhexadecylammonium chloride (BDAC) to cetyltrimethylammonium bromide (CTAB) growth solution to prepare Au nanorods with adjustable aspect ratio [91]. Adding organic additives such as sodium salicylate and sodium oleate to CTAB growth solution can also change the micellar behavior of CTAB. In this way, the near-infrared spectrum of Au with an adjustable aspect ratio and good monodispersity can be obtained. In addition, they proved that in the growth solution of the binary surfactant system, the concentration of didodecyldimethylammonium bromide (DDAB) plays an important role in changing the shape and aspect ratio of Au nanoparticles. Au-NRS can be obtained at low DDAB concentrations. The increase of DDAB concentration promoted the formation of Au biconical structure. However, due to the cytotoxicity of cetyltrimethylammonium bromide used in the synthesis process, it will hinder the application of AuNRs in the field of biological sciences [92]. In order to overcome these obstacles, customized ligands to AuNRs, for example, combined with polyethylene glycol (peg), can effectively reduce cytotoxicity [93].

**Figure 3:**
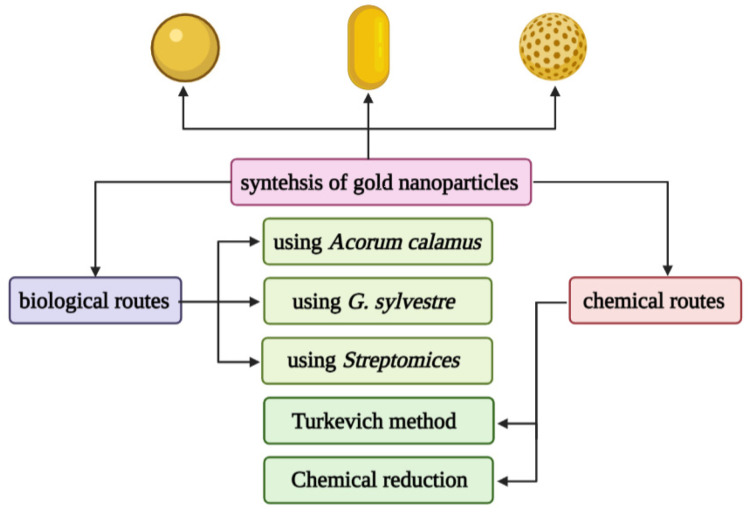
Different chemical and biological synthesis methods of gold nanoparticles.

Gold nanoshells (AuNSs) are spherical structures, including a silicon core and a thin gold layer, with a size between 50–150 nm [94]. Their optical properties can be adjusted by changing the core diameter and the thickness of the shell. AuNSs has great potential in the imaging and treatment of cancer patients. AuNSs has been proven to use imaging to detect cancer, such as two-photon induced photoluminescence, diffuse reflectance optical tomography, X-ray imaging, and photoacoustic imaging [95]. On the other hand, due to their hollow structure, AuNSs show interesting properties as drug carriers. It is well known that EphB4 is overexpressed in several tumor types, such as breast cancer, prostate cancer, bladder cancer, lung cancer, colon cancer, gastric cancer, and ovarian cancer. You prepared HAuNS-doxorubicin by its binding peptide with ephb4 (T-HAuNS-doxorubicin) [96]. The resultant T-HAuNS-doxorubicin showed selective uptake in mouse ovarian cancer xenografts, and its tumor uptake rate was 50–100% higher than its non-targeted counterparts. It also shows excellent efficacy in anti-tumor treatment. The anti-tumor effect in vivo shows that the tumor can disappear after using T-HAuNS-doxorubicin. The main synthesis method of gold nanoparticles is shown in Figure 3.

## 5. Metal Nanoparticles and PDT

Currently, significant progress has been made in tumor immunotherapy. However, the complex tumor microenvironment (TME) prevents activated immune recognition of cancer cells [97]. Tumor tissue hypoxia has always been an obstacle to the clinical application of PDT. Besides, the low oxygen partial pressure of TME also reduces the power of PDT [98].

In recent years, metal nanoparticles have attracted much attention due to their unique physical (such as plasmon resonance and fluorescence enhancement) and chemical (such as enhanced catalytic activity) properties [99]. Because metal nanoparticles have a large specific surface area and area: volume ratio, they exhibit several special physicochemical properties, making them useful for cancer treatment. As therapeutic agents, metal nanoparticles are more advantageous than other nanoparticles due to their inherent anti-cancer activity, which overcomes the requirements of other carriers for therapeutic and diagnostic drug delivery [100]. In addition, they are biocompatible in nature and can be easily excreted from the body. At the same time, the therapeutic moieties can also be wrapped or conjugated with metal nanoparticles, thus, the metal nanoparticles can also be used as a carrier for the therapeutic moieties. By conjugating a specific targeting moiety to the surface of the metal nanoparticle, the surface of the metal nanoparticle can be modified to target cancer cells specifically. Moreover, due to the inherent properties of metal nanoparticles, they are also used for diagnostic and imaging purposes [101,102]. For example, the photoluminescence or superparamagnetic properties of metal nanoparticles are useful for imaging. Thus, as therapeutic agents, metal nanoparticles provide a means of cancer diagnosis, treatment, and monitoring within one formulation, and ultimately improve the efficacy of anti-cancer treatment. This can also reduce patient inconvenience and potential adverse effects.

**Figure 4:**
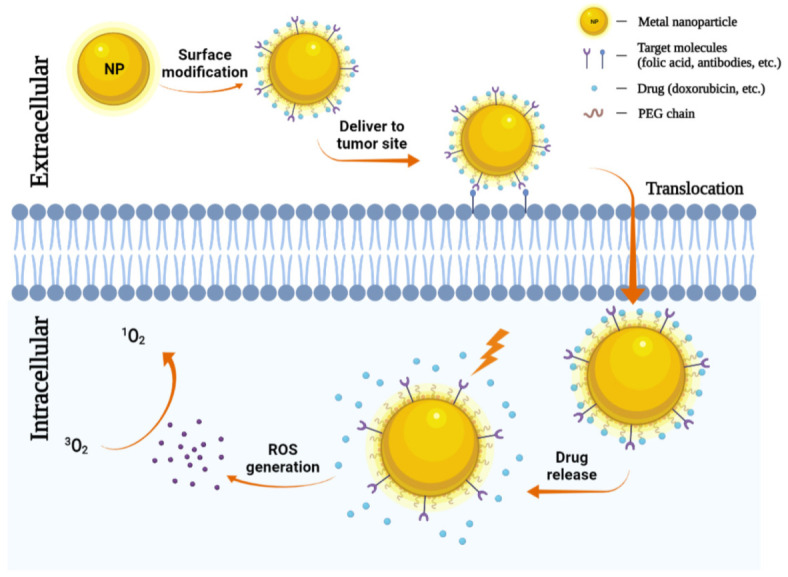
Metal nanoparticles (NPs) in chemotherapy. NPs can carry antibodies against tumor-specific receptors thereby driving NPs to the tumor site. NPs penetrate the plasma membrane for the release of chemotherapeutic agents. Then under a specific wavelength of light, they can generate ROS and induce tumor cell death.

The use of nanoparticles as delivery systems for both photosensitizers and chemotherapeutics at the same time has also been attracted more attention. Selective accumulation of PSs in tumors is critical for effective PDT by minimizing collateral damage to surrounding healthy tissues. However, typically PSs are hydrophobic and aggregate in aqueous media, which deleteriously affect their photophysical (decreased ^1^O_2_ formation), chemical (decreased solubility), and biological (insufficient tumor localization) properties, thereby diminishing the PDT efficacy [33]. Nanoparticles can increase the solubility of hydrophobic therapeutic or PDT agents and offer proper size and surface properties to prolong blood circulation, allowing for their selective accumulation in tumors via the enhanced permeability and retention (EPR) effect [103,104]. Tumor accumulation may be further improved by modifying the particle surface with cancer-targeting ligands. Indeed, several nanoparticles have been explored as promising delivery vehicles for a molecule or material-based PDT alone or combined with chemotherapeutic agents to cancers to enhance the photo treatment efficiency, and in some cases, encouraging preclinical and clinical data are emerging [105,106]. The role of metal nanoparticles in chemotherapy-photodynamic therapy is shown in Figure 4.

## 6. Combination of PDT with Other Therapies

PDT alone for breast cancer is far from clinical cancer treatment. Therefore, PDT should be used as a salvage and palliative measure for comprehensive cancer treatment to achieve an optimal treatment effect. Correctly, it plays a vital role in combination with chemotherapy, immunotherapy, and targeted therapy for the treatment of breast cancer.

### 6.1. PDT Combined with Chemotherapy

Chemotherapy is one of the main treatments for breast cancer, usually in the form of a combination of drugs to maximize tumor eradication without poisoning any host system [107]. The antitumor mechanism of chemotherapeutic drugs is generally believed to bind to the DNA of tumor cells and thereby prevent DNA replication. These chemotherapeutic drugs kill cancer cells to some extent, while they also have severe side effects on the whole body because of their non-specificity [108]. To improve the therapeutic effect and overcome drug resistance, many attempts have been made to combine PDT with chemotherapy [109].

Combining anti-cancer drugs with suitable nano-carriers can prolong the half-life of the drug in the body and control the release process of the drug. The ideal drug carrier should have special characteristics, such as chemical stability, long half-life, non-toxic or low-toxicity, biodegradability, and high binding capacity [110]. In particular, functionalized metal nanostructures with the ability to encapsulate or bind drugs have more advantages than free drugs and have the advantages of lower drug toxicity, higher targeting efficiency, and longer half-life, which are used to overcome the deficiencies of chemotherapy and photodynamic therapy [109,111].

Among many nano-scale materials, hollow gold nanoparticles (HAuNP) have become an excellent candidate for drug delivery due to their cavity structure, high availability of internal and external surfaces, good biosafety, biocompatibility, and functional capabilities [112]. The surface plasmon absorption band of HAuNP can be adjusted by adjusting the inner diameter and thickness of the gold shell from visible light to near-infrared. Due to the unique physical properties and low toxicity of HAuNP, they are widely used in medical nanotechnology. In Armin Imanparast’s research, HAuNP was used to encapsulate mitoxantrone (MTX) and used light-emitting diodes (LEDs) to irradiate different amounts of radiation at different concentrations of therapeutic doses [113]. Studies have found that the PEGylation of HAuNP increases the amount of nanoparticles uptake by cells and improves the binding capacity of MTX. Luyun Zhang constructed AuNCs and metal organic framework (MOF) wrapped doxorubicin (DOX) and ZIF-8, and synthesized them into pH-responsive multifunctional nanoprobes for simultaneous PDT/chemotherapy [114]. Among them, AuNCs are encapsulated into the internal structure of ZIF-8, and DOX is loaded into the channel of ZIF-8. Due to the encapsulation of ZIF-8, AuNCs and DOX are not released in a neutral medium, avoiding the threat to normal organizations. More importantly, due to the collapse of the pH-responsive structure of ZIF-8 in acidic media, the release of AuNCs and DOX in tumor cells is accelerated, thereby enhancing the performance of breast cancer PDT and chemotherapy. In Weijun Xu’s research, he first modified GNRs with mercaptopropionylhydrazine (MPH) [115]. Subsequently, the chemotherapeutic drugs doxorubicin (DOX) and pro-photosensitizer ALA were linked to MPH through hydrazone bonds, forming the nanoplatform GNRs-MPH^-ALA/DOX^-PEG, which was used for the pH response drug release and CT/PDT/PTT triple therapy of breast cancer. GNRs-MPH^-ALA/DOX^-PEG can exhibit excellent dispersibility and colloidal stability in physiological solutions and has excellent light-to-heat conversion efficiency. DOX and ALA bind to GNRs through hydrazone bonds, ensuring pH-responsive drug release. MCF-7 cells can effectively take up the nanomaterial, and DOX can effectively enter the nucleus. Under near-infrared light irradiation, the nano-platform can effectively generate heat for PTT and sufficient active oxygen for PDT. It proved the superadditive effect of CT/PDT/PTT triple combination therapy. Triple therapy can completely inhibit tumor progression without obvious side effects on normal tissues. In short, the simple nano-platform GNRs-MPH^-ALA/DOX^-PEG can be used as an ideal nano-platform for high-efficiency triple therapy of breast cancer.

### 6.2. PDT Combined with Immunotherapy

In recent years, immunotherapy has attracted much attention due to its inhibitory effect on distant and metastatic tumors [116]. Immunotherapy is considered to be a potentially effective way to defeat cancer by regulating the systemic immune system rather than focusing on the tumor itself [117]. Many immunologically active nanomedicines have been developed to effectively deliver antigens or viral peptides to antigen-presenting cells and further stimulate the response of memory T cells to tumors [118]. The premise of tumor immunotherapy is that tumor cells can be eliminated by host cytotoxic CD8+ T cells, and these cells themselves can be affected by regulatory T (Treg) cells, induced expression of programmed death-1 (PD-1) and other inhibitory checkpoint receptors, which limit the antitumor function of cytotoxic lymphocytes [119]. The immune response caused by PDT plays a vital role in preventing metastasis and recurrence of the tumor [120]. The mechanism of these processes is complex and involves almost all aspects of the immune system. To achieve better therapeutic effects, it is necessary to combine immunotherapy with other therapies. The combination of phototherapy and immunotherapy provides a new way to treat tumors [121,122]. Under the light, the heat or active oxygen generated by phototherapy can kill cancer cells, and the immunogenic cell death (ICD) induced by phototherapy can enhance anti-tumor immunity. ICD releases impaired related molecular patterns (DAMP), which leads to an increase in the immunogenicity of the tumor microenvironment. Many nanomaterials have been prepared for synergistic phototherapy and immunotherapy [123,124,125].

In this regard, Xiaopin introduced a promising strategy for the treatment of primary in vitro 4T1 BC tumors using immunotherapy based on checkpoint blockade [126]. Use zinc and pyrophosphate (ZnP) to make a non-toxic core-shell composed of ZnP@pyro NPs, and incorporate tar PS into its core for PDT applications, and add PD-L1 antibody for checkpoint blocking immunity Treatment. According to research results, immunogenic ZnP@pyro NPs are non-toxic before photoactivation. Moreover, it could be effectively inhibited without distant metastasis by inducing a potent antitumor immune response. In the PDT study, they successfully eliminated BC cells in vitro through apoptosis and necrosis under 670 nm irradiation. The in vivo PDT study of ZnP@pyro NPs on 4T1 tumor-bearing mice in situ shows that this immunogenic PS nanocarrier enhances PS uptake through the EPR effect to achieve high tumor accumulation, as well as destroy the tumor vascular system and increase tumor immunogenicity. As a result, Znp@pyro not only eradicated the primary tumors but also prevented lung metastasis and inhibited the preexisting metastatic tumors by generating systemic antitumor immunity.

### 6.3. PDT Combined with Targeted Therapy

PS subcellular localization uptake can be divided into passive targeting and specific active targeting. Passive PS uptake is caused by the permeability and retention effect, which can lead to vascular leakage in tumor tissues [127]. This is a naturally occurring process that uses differences in anatomical and pathophysiological abnormalities between cancer tissues and normal cells to improve PS inactivation in tumor cells. When nanoparticle carriers are combined with PS, they often promote the passive absorption of PSs through the EPR effect [128]. Active targeting requires specific targeting ligands (such as antibodies, small ligands, peptides, folic acid, or carbohydrates) to bind to the surface of the PS-loaded nanocarrier system [129,130,131]. Therefore, PS uptake in these cells is specifically enhanced. Compared with passive targeting, active nanoparticle targeting can enable tumor cells to absorb PS more selectively, making the concentration of nanocarriers and drugs in tumor cells higher.

Recently, a multi-stimulus response therapeutic diagnostic nano-platform was proposed [132]. The nanoplatform is based on functionalizing AuNR with hyaluronic acid (HA), and then binding anti-HER-2 antibody, 5-aminolevulinic acid (ALA), and Cy7.5 to HA to enhance active PDT targeting and fluorescence, respectively Imaging. The cell uptake efficiency of AuNR-HA^-ALA/Cy7.5^-HER-2 showed that compared with the control group, the uptake of MCF-7 cells was significantly increased by 75.5%, indicating that the nanoplatform was mediated by specific HER-2 receptors. The guided dual-targeting strategy improves PS absorption. In addition, PDT MCF-7 cells treated with AuNR-HA^-ALA/Cy7.5^-HER-2 alone at 635 nm reported a cell viability reduction of 75.6%, while cells treated with a single PTT at 808 nm showed a cell viability reduction of 58.4%. Overall, the combined PDT/PTT mode of 5.5 µg/mL ALA concentration and AuNR-HA^-ALA/Cy7.5^-HER-2 nanoplatform noticed a significant 61.2% cell death. Magnetic NPs (MNPs) have high-field irreversibility, small size, and surface function, so they have attracted great attention in vivo and in vitro biomedicine [133,134]. In therapeutic research, they can promote drug targeting and assist in diagnostic applications [135]. In the study by Matlou to evaluate the PDT activity of two zinc phthalocyanine derivatives, zinc monocinnamic acid phthalocyanine, and zinc mono-carboxy phenoxy phthalocyanine complexes were combined with folic acid (FA) targeting agents, and amino-functionalized Fe_2_O_3_ MNP (AMNP) is covalently linked [136]. The dark toxicity of this MNP PS carrier is significantly reduced after FA complex attachment, and the effect of Pc-AMNP on MCF-7 PDT in vitro shows that it can kill 60% of tumor cells under 670 nm irradiation. The research of Feng adopted a promising strategy called “integration”, which allows dual imaging-guided PDT in MCF-7 BC in vitro by anchoring PS to UCNP, thereby combining imaging and therapeutic The PDT function is integrated into a nano-platform cell [137]. In this study, NaYF_4_: Er, Yb@NaYF_4_ UCNP was synthesized and covalently combined with pre-targeted tetrazine (Tz) and FA molecules to form UCNPsTz/FA-PEG, which was used as a bio-orthogonal reaction in deep tumor tracking and imaging. Then when RB-NB PS is attached to the surface of the nanoplatform through a bio-orthogonal chemical reaction, it shows effective PS targeting. Due to the active targeting of FA and the EPR effect, upconversion luminescence (UCL) imaging of nude mice injected with MCF-7 BC cells showed a high accumulation of nanoplatforms in tumor sites. In addition, when the in vivo PDT analysis of these tumor-bearing mice was treated with NPs-Tz/FA-PEG RB-NB under 980 nm irradiation, the tumor size was reduced by 75.5% compared with the control group.

## 7. Conclusions

BC is aggressive cancer that can metastasize and often recur after treatment [138]. Many conventional therapies for BC often exhibit some form of resistance and unwanted side effects, and surgery is invasive [139]. In this sense, PDT is gaining a prominent position as a non-invasive, highly targeted treatment. However, twenty years after photosensitizers were first approved for PDT, this therapy has not yet entered the central stage of oncology. At present, how to maximize the accumulation of drugs in tumor sites and reduce systemic side effects are still the main challenges faced by PDT researchers.

The combination of NPs and PSs to passively and more selectively enhance their accumulation in tumor tissues to improve PDT treatment results and reduce unnecessary side effects on local tissues is rapidly becoming a popular method [127].

Metal NPs have unique properties that help reduce PS leaching, allow high load capacity of PSs, improve passive PS absorption through the EPR effect, and allow easy functionalization with various ligands to promote active PS absorption, thereby allowing the overall enhanced PDT BC processing [22,140].

In addition, compared with organic NPs, metal PS nanocarriers are not easily degraded and will not release the attached PSs, but allow the activated ROS to diffuse out after irradiation, so they are more significantly used in the PDT field [140]. In addition, new ways to improve the effectiveness of PDT are constantly being discovered. Regarding the combination of PDT and other forms of anti-cancer therapy, a large amount of preclinical evidence has been collected, and it seems to be increasing rapidly [141,142]. Especially promising is the combination of PDT with chemotherapy, immunotherapy, and targeted therapy. The in-depth understanding of the biological mechanism of PDT provides fertile soil for the cultivation methods to improve the effects of various related treatments. By constructing nanoparticles that can meet the complex tumor microenvironment and regulate the breast cancer tumor cell microenvironment, the drug delivery effect can be enhanced, and the systemic effects of chemotherapy and immunotherapy can be enhanced. A large number of studies highlighted in this review indicate that the potential of using PDT as part of combination therapy is being investigated from many different directions, with promising results in terms of direct cytotoxicity and promotion of a strong anti-tumor immune response. In addition, anchoring the active targeting moiety to the metal NP loaded with PS allows the nanosystem to target only BC cells, thereby enhancing PS accumulation, limiting unnecessary damage to normal cells, and promoting PDT treatment results [127,143]. However, determining the strategy of using combined applications requires further research and multidisciplinary cooperation to enhance the overall PDT treatment model for breast cancer.

Taking into account the above aspects, photodynamic therapy has become more and more prominent among clinicians and patients. The rapid development and advancement of technology and optoelectronic equipment are conducive to the application of photodynamic methods in clinical practice. It is speculated that shortly, the application of nanotechnology to enhance photodynamic therapy should be able to overcome breast cancer [143]. However, more comprehensive studies are needed to carefully examine the long-term toxicity, pharmacokinetics, and pharmacodynamic properties of nanocarriers in order to achieve maximum accumulation and PS uptake in target tissues. In addition, although anchoring PSs on the surface of nanoparticles can improve their biocompatibility, its potential toxicity and unnecessary liver and kidney accumulation must also be considered. Therefore, the metal NP research discussed above must be further studied in clinical trials. It is hoped that great progress has been made in systemic anti-tumor protection and the potential to combine PDT with other treatment strategies to make PDT treatment of breast cancer a reality in the future.
